# Fucoidan Stimulates Monocyte Migration via ERK/p38 Signaling Pathways and MMP9 Secretion

**DOI:** 10.3390/md13074156

**Published:** 2015-06-30

**Authors:** Elene Sapharikas, Anna Lokajczyk, Anne-Marie Fischer, Catherine Boisson-Vidal

**Affiliations:** 1 Inserm UMR_S 1140, Faculté de Pharmacie, Université Paris Descartes, Sorbonne Paris Cité, 4 Avenue de l’observatoire Paris 75006, France; E-Mails: anna.lokajczyk@parisdescartes.fr (A.L.); catherine.boisson-vidal@parisdescartes.fr (C.B.-V.); 2 Inserm UMR-S 970, AP-HP, Hôpital Européen Georges Pompidou, 20 rue Leblanc Paris 75015, France; E-Mail: anne-marie.fischer@egp.aphp.fr

**Keywords:** fucoidan, monocytes, critical limb ischemia, migration

## Abstract

Critical limb ischemia (CLI) induces the secretion of paracrine signals, leading to monocyte recruitment and thereby contributing to the initiation of angiogenesis and tissue healing. We have previously demonstrated that fucoidan, an antithrombotic polysaccharide, promotes the formation of new blood vessels in a mouse model of hindlimb ischemia. We examined the effect of fucoidan on the capacity of peripheral blood monocytes to adhere and migrate. Monocytes negatively isolated with magnetic beads from peripheral blood of healthy donors were treated with fucoidan. Fucoidan induced a 1.5-fold increase in monocyte adhesion to gelatin (*p* < 0.05) and a five-fold increase in chemotaxis in Boyden chambers (*p* < 0.05). Fucoidan also enhanced migration 2.5-fold in a transmigration assay (*p* < 0.05). MMP9 activity in monocyte supernatants was significantly enhanced by fucoidan (*p* < 0.05). Finally, Western blot analysis of fucoidan-treated monocytes showed upregulation of ERK/p38 phosphorylation. Inhibition of ERK/p38 phosphorylation abrogated fucoidan enhancement of migration (*p* < 0.01). Fucoidan displays striking biological effects, notably promoting monocyte adhesion and migration. These effects involve the ERK and p38 pathways, and increased MMP9 activity. Fucoidan could improve critical limb ischemia by promoting monocyte recruitment.

## 1. Introduction

Cardiovascular disease is the leading cause of death worldwide. Peripheral arterial disease (PAD) is linked to a three- to six-fold increase in cardiovascular mortality compared to the general population [1,2,3]. With population aging, PAD has become a major public health problem [4]. Revascularization currently relies on bypass surgery or endovascular therapy (balloon angioplasty or stents) [2,5,6]. Conservative surgery is not always possible, and the affected limb must sometimes be amputated to avoid necrosis [7]. PAD is initially asymptomatic, and its diagnosis is based mainly on the ankle brachial pressure index. However, media sclerosis can interfere with this index [8], especially in older people and patients with diabetes, further delaying diagnosis and treatment in some cases [9]. Current treatments do not always avoid limb amputation or death [10,11,12]. Great hopes are being placed in gene and cell therapies. However, a large randomized placebo-controlled phase III trial in critical limb ischemia, the TAMARIS study, showed no reduction in the amputation rate in patients treated with a plasmid encoding acidic FGF (fibroblast growth factor) [13], thus failing to confirm benefits seen in phase II trials [14,15]. Protective effects have been observed with other angiogenic growth factors (FGF2 and VEGF) [16,17,18]. Several studies have shown an improvement in patients’ health status after intramuscular injection of bone marrow- or peripheral blood-derived mononuclear cells [19,20,21]. However, none of these trials showed efficient revascularization [22,23]. Endothelial progenitor cells are mononuclear cells involved in vascular and tissue remodeling. Several studies have shown the direct beneficial involvement of monocytes in PAD [24,25]. In particular, the early presence of monocytes at ischemic sites resulted in increased reperfusion in a murine model of lower limb ischemia [26]. Mobilization and recruitment of circulating monocytes from bone marrow to sites of active revascularization, where they differentiate into macrophages, is crucial for tissue regeneration after an ischemic event. The first step of monocyte recruitment involves tethering and rolling along the vessel endothelium, followed by strong adhesion and tissue entry*.* Several studies have shown an important role for monocyte chemoattractant protein-1 (MCP1) and its receptor CCR2 in monocyte mobilization at ischemic sites. Inhibition of this recruitment negatively affects the angiogenic process, as demonstrated in the CCR2 knock out mouse model [27,28]. During PAD, increased MCP-1 secretion leads to monocyte recruitment and is involved in the angiogenic process. Voskuil *et al*. showed that MCP-1 injection after femoral artery ligation in pigs stimulated collateral vessel formation [29].

Our laboratory studies a low-molecular-weight (LMW) sulfated polysaccharide extracted from brown seaweeds. Fucoidan exhibits exceptional enhancement of new blood vessel formation in animal models [30,31,32,33]. LMW fucoidan enhances the proangiogenic properties of endothelial colony-forming cells (ECFC) *in vitro*, by modifying both early events (proliferation and migration) and late events (differentiation into vascular cords) [31]. In a previous study, we showed that fucoidan significantly improved the beneficial effects of ECFC transplantation in a mouse model of hind limb ischemia, preventing tissue necrosis [30]. This tissue protection was associated with enhanced neoangiogenesis and a reduction in rhabdomyolysis. Fucoidan prestimulation enhanced each step of the angiogenic processes, namely cell recruitment to ischemic tissue *via* enhanced ECFC adhesion to activated endothelium, MMP-9 secretion, extravasation, and differentiation into a vascular network. In the present study, we investigated the mechanism of action of fucoidan on peripheral blood monocyte cells (PBMC) adhesion to gelatin and migration through an activated endothelium, as well as adhesion molecule expression and the MMP2/MMP9 secretion. We also explored the signaling pathway involved in fucoidan-induced monocyte migration.

## 2. Results and Discussion

### 2.1. Fucoidan Pretreatment Enhances Monocyte Adhesion, Migration and Transmigration

**Figure 1:**
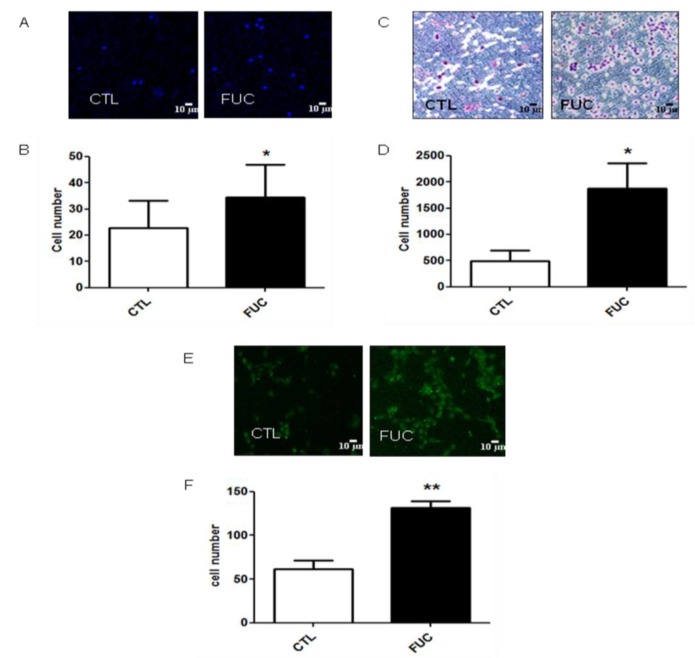
Fucoidan enhances monocyte adhesion to gelatin, and their migration. (**A**) Representative results obtained with PBMC after 30 min, with or without 24 h of fucoidan pretreatment; (**B**) Monocyte adhesion (in white, control monocytes, in black, monocytes incubated with 10 μg/mL fucoidan; (**C**) Representative results for migration of isolated PBMC treated with or without fucoidan (4 h) towards 100 ng/mL MCP-1; (**D**) Migratory cell numbers in five independent fields; (**E**) Representative monocyte transmigration (18 h) with or without fucoidan pretreatment (30 min); (**F**) Transmigratory cell numbers in five independent fields. Three to five independent donors. * *p* < 0.05; ** *p* < 0.01.

The first step of monocyte recruitment is their adhesion to the endothelium, followed by migration and transmigration through the endothelium. We first investigated the effect of fucoidan on monocyte adhesion to gelatin (Figure 1A). Fucoidan treatment for 24 h enhanced monocyte adhesion by 1.5-fold (Figure 1A,B, *p* < 0.05). As shown in Figure 1C, fucoidan enhanced PBMC migration in a concentration-dependent manner (data not shown). Monocytes pretreated with fucoidan were 5-fold more motile than control cells towards 100 ng/mL MCP-1 (*p* < 0.05, Figure 1D). As shown in Figure 1E, pretreatment of PBMC with fucoidan led to a 2.5-fold increase in transmigration across an activated monolayer of HUVEC (Figure 1F, *p* < 0.01). These results showed that, *ex vivo*, fucoidan enhanced all the major steps of monocyte recruitment to ischemic sites.

### 2.2. Fucoidan Stimulation of Monocyte Adhesion and Migration Is not Due to Modulation of Integrin Expression or CCR2 Receptor Expression

**Figure 2:**
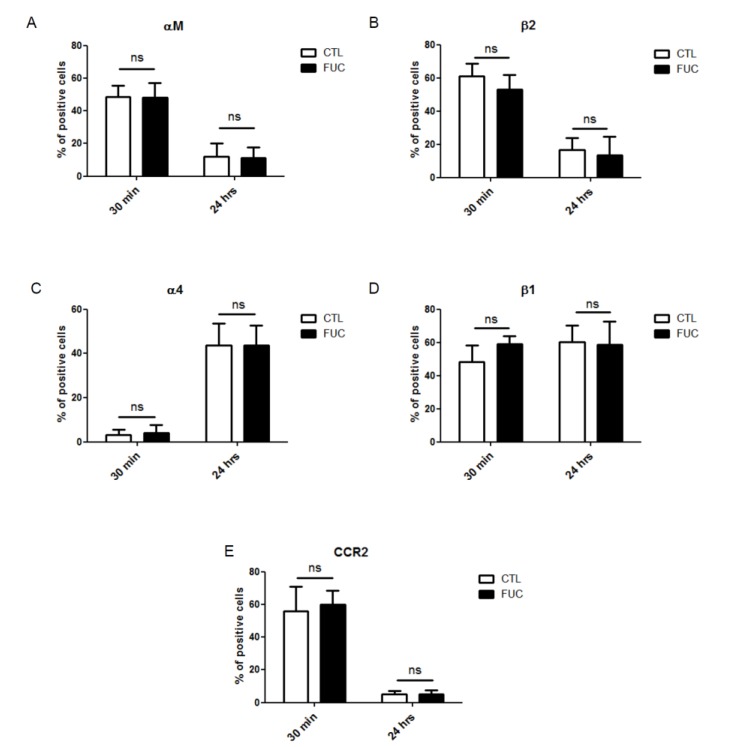
Impact of fucoidan on adhesion molecule and CCR2 receptor expression: PBMC were treated for 30 min or 24 h with (black bars) or without fucoidan (white bars). (**A**) Percentage of monocytes positive for alpha M expression; (**B**) Percentage of beta 2-positive cells; (**C**) Percentage of alpha 4-positive cells; (**D**) Percentage of beta 1-positive cells; (**E**) Percentage of CCR2-positive cells (4 independent donors).

As fucoidan-treated PBMC showed a striking increase in adhesion and migration, we examined whether fucoidan modulated the expression of integrins involved in these processes. As engagement of monocyte integrins αMβ2 (VLA4) and α4β1 (MAC-1) by endothelial ICAM-1 and VCAM, respectively, is critical for monocyte extravasation, we examined the effect of fucoidan on the expression levels of these integrins after 30 min or 24 h of fucoidan exposure. As shown in Figure 2, neither exposure time affected PBMC integrin expression (Figure 2A–D). MCP-1 receptor (CCR2) expression was not modulated by fucoidan after 20 min of incubation (Figure 2E). Surprisingly, however, CCR2 expression was downregulated after 24 h, in the presence or absence of fucoidan (Figure 2E), possibly because CCR2 is involved in the early phase of recruitment of monocytes, whereas CXCR1 takes over during the second phase [34,35]. Finally, fucoidan did not affect the expression of CD44 or CD87, two receptors involved in monocyte migration and actin cytoskeleton rearrangement involved in cell motility (data not shown).

### 2.3. Fucoidan Enhances PBMC MMP9 Activity

**Figure 3:**
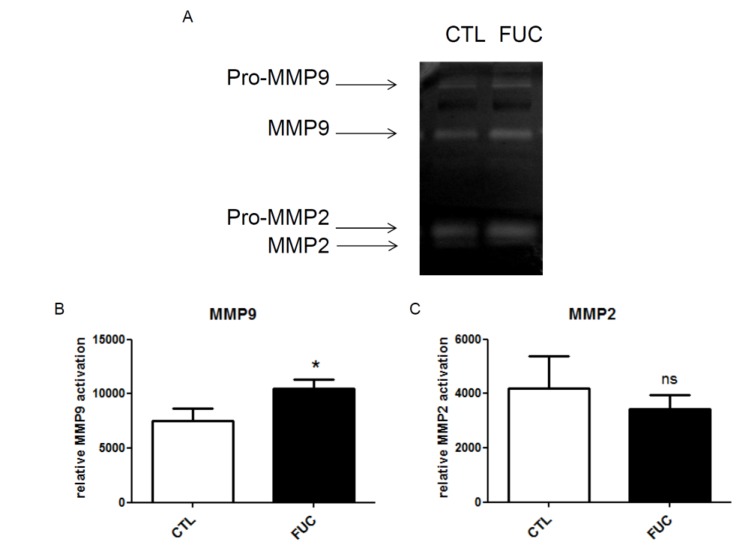
Impact of fucoidan on monocyte MMP9 expression (gelatinolytic activity): (**A**) Representative gelatin zymography of culture supernatant of monocytes treated with or without fucoidan for 30 min; (**B**) MMP9 gelanitolytic activity; (**C**) MMP2 gelatinolytic activity. Three independent donors; * *p* < 0.05

As fucoidan-treated monocytes showed no change in integrin or CCR2 receptor expression, we explored the possible role of matrix metalloproteinases in fucoidan-enhanced migration and adhesion. Extracellular MMPs are involved in monocyte migration: macrophage adhesion to fibronectin via α5β1 integrin *in vitro* is associated with increased MMP9 secretion [36]. Furthermore, we have shown that fucoidan increases MMP9 activity in HUVEC and ECFC cells [30,31]. Here, MMP9 and MMP2 activities were quantified by gelatin zymography (Figure 3A). We observed a significant increase in MMP9 activity in conditioned media of fucoidan-treated PBMC (Figure 3B, *p* < 0.05). MMP2 activity was unaffected (Figure 3C). This effect of fucoidan on MMP9 secretion is unlikely to be sole mechanism underlying the observed effect of fucoidan on monocyte migration and adhesion.

### 2.4. Fucoidan Enhancement of PBMC Migration Is Countered by ERK and p38 Pathway Inhibition

**Figure 4:**
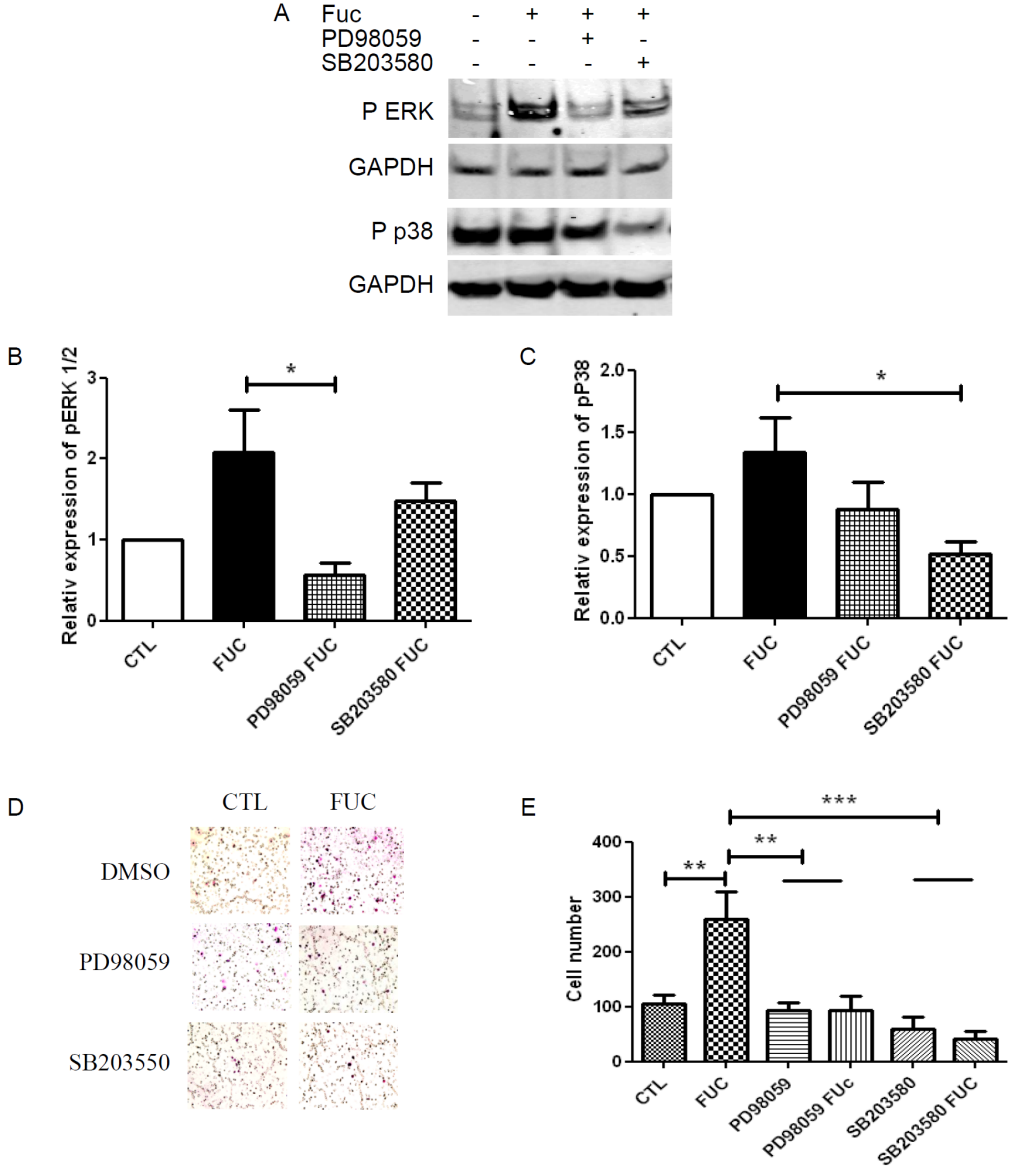
ERK and p38 signaling pathway involvement in fucoidan-treated monocyte migration: (**A**) Representative Western blot illustrating phosphorylation of ERK1/2 and P38 when PBMC were treated with or without fucoidan (in the presence or absence of PD98059 or SB203580) for 30 min; (**B**) Quantitative analysis of ERK phosphorylation; (**C**) Quantitative analysis of p38 phosphorylation. Results are represented relative to the corresponding control, with with independent donors; (**D**) Representative fields showing migratory cells treated as in A; (**E**) Migratory cell numbers in five independent fields. * *p* < 0.05; ** *p* < 0.01; *** *p* < 0.001 compared to control. Four independent donors.

The MAPK ERK and p38 pathways have been shown to be involved in monocyte migration. We used Western blot to analyze the phosphorylation levels of ERK1/2 and p38 in starved and re-stimulated monocytes, treated with or without fucoidan, in the presence of specific inhibitors of these kinases (Figure 4A). Fucoidan-treated monocytes showed a two-fold increase in ERK phosphorylation, and this increase was inhibited by the ERK inhibitor PD98059 (Figure 4B). Fucoidan treatment also increased p38 phosphorylation to a lesser extent, an effect also inhibited by the p38-specific inhibitor SB203580 (Figure 4C). Finally, we explored the role of the ERK and p38 pathways in PBMC migration towards MCP-1 (Figure 4D). As expected, ERK and p38 inhibition abrogated the ability of fucoidan to enhance monocyte migration. Although neither pathway seemed to be involved in monocyte migration nor in control conditions, ERK and p38 inhibition reduced the migration of fucoidan-treated monocytes by 2.5-fold (Figure 4E), highlighting the prominent role of these pathways in fucoidan-enhanced monocyte migration.

## 3. Discussion

**Figure 5:**
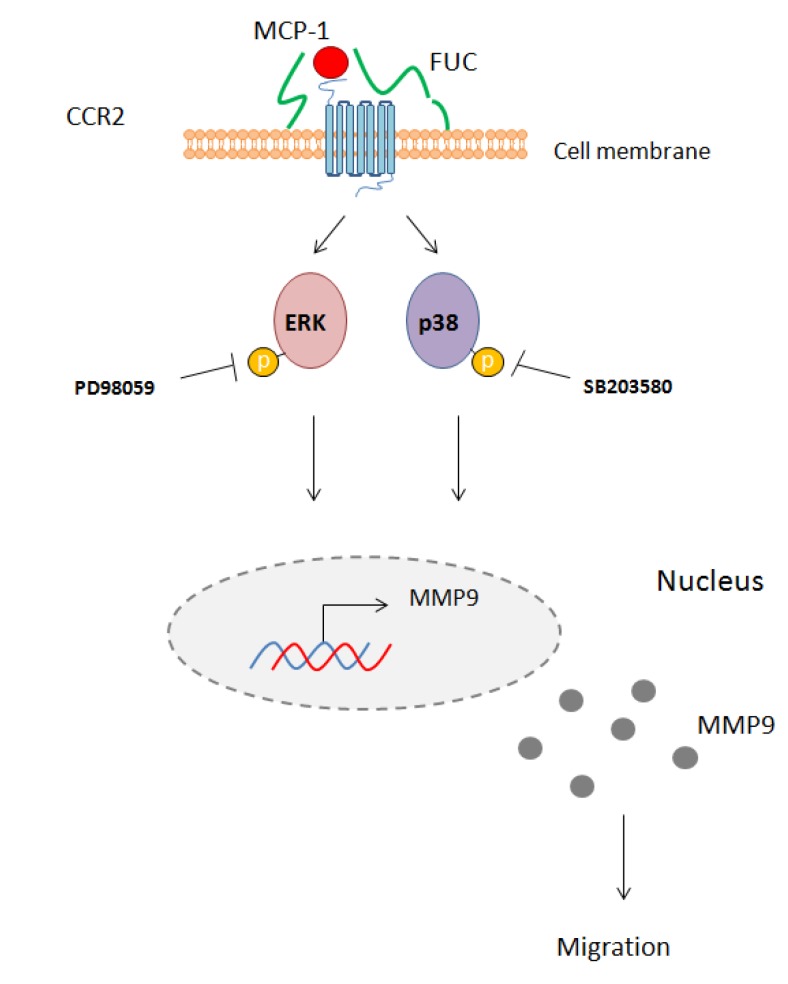
Schematic overview of the effect of fucoidan on monocyte migration. Fucoidan bound to the cell membrane enhances MCP-1 interaction with its receptor CCR2. This interaction leads to phosphorylation of ERK1/2 and p38 and activates MM9 secretion. PD98059 and SB203580 inhibit this phosphorylation, leading to reduced monocyte migration.

We provide new evidence for a major effect on fucoidan on monocyte migration. We found that fucoidan did not modulate integrin or receptor expression on the monocyte cell membrane. However, fucoidan enhanced monocyte migration towards MCP-1, an effect associated with ERK and p38 signaling pathway activation and with MMP9 secretion (Figure 5).

In response to diverse pro-inflammatory signals released from damaged tissue, circulating blood monocytes attach transiently to the activated vascular endothelium and resist shear stress before crossing the vessel wall [37]. Several studies have highlighted the importance of monocyte recruitment for tissue and vessel repair [28,38,39]. Here we show that PBMC pretreatment with fucoidan enhanced their adhesion to gelatin. Elsewhere, fucoidan-treated ECFC have been found to adhere more efficiently to activated endothelium in flow conditions [30]. We also found that fucoidan enhanced monocyte migration towards MCP-1 as chemoattractant, and also favored monocyte transmigration on a monolayer of activated endothelial cells. This increased response of PMBC to MCP-1 may have therapeutic relevance, as this chemoattractant has been shown to be involved in monocyte recruitment, particularly during neovessel formation. Vein graft intimal hyperplasia is associated with MCP-1 upregulation, leading to monocyte recruitment. Furthermore, macrophage depletion with liposome clodronate diminishes MCP-1 and TGF beta 1 expression, an effect associated with reduced vein graft healing in rats [40]. Schepers and collaborators confirmed these results with anti-MCP-1 in mice, as did Tatewaki *et al*. using adenoviral gene transfer to block MCP-1 expression in dogs [41,42]. Our results indicate that fucoidan promotes the early phase of monocyte recruitment to activated endothelium and, subsequently, new vessel formation.

The precise mechanism of action of fucoidan on PBMC is not fully understood. We have previously demonstrated that GAG abrasion on the cell surface hinders ECFC migration, and that fucoidan treatment restores this migration [30]. Like glycosaminoglycans, fucoidan, by its ionic structure, is able to bind adhesion proteins [43], growth factors [44] and cytokines [45]. The activity of fucoidan is due mainly to its sulfatation: desulfated fucoidan loses its proangiogenic properties *in vitro* and *in vivo,* and is unable to recruit hematopoietic stem cells [46]. As fucoidan interacts with adhesion proteins, we examined whether fucoidan treatment enhanced PBMC expression of integrins involved in monocyte migration. Fucoidan had no effect on the expression of integrins or CCR2, the main MCP-1 receptor. Interestingly, αM/β2 integrin expression was reduced after 24 h of culture, while α4 integrin expression was increased, but these changes occurred irrespective of fucoidan treatment.

Fucoidan treatment increased the phosphorylation of ERK 1/2 and p38, two signaling pathways involved in monocyte migration and transmigration [47,48]. Surprisingly, we found that these two signaling pathways were not involved in monocyte chemoattraction *ex vivo*, as their inhibition did not inhibit the migration of monocytes not treated with fucoidan. This discrepancy with previous reports may be explained by the use of different models, as most published studies used monocytic cell lines such as THP1. Ashida *et al*. reported that the ERK pathway is involved in monocyte adhesion, while the p38 pathway would be involved in cell migration [49]. In contrast, and in accordance with our findings, it has been shown that the ERK and p38 pathways are not involved in the migration of fresh PBMC [50]. Our results support a direct role of ERK and p38 in fucoidan-enhanced monocyte migration. Indeed, inhibition of either pathway abolished the effect of fucoidan. Finally, we found that fucoidan enhancement of *ex vivo* monocyte migration was associated with MMP9 secretion. It has been shown that monocyte migration is specifically associated with MMP9 activity, through ERK activation [48,51]. Fucoidan was also reported to be an antitumor compound inhibiting migration, invasion and MMP-2/-9 activities in human fibrosarcoma cells (HT1080), human lung cancer cells (A549) and mouse hepatocarcinoma cells lines (Hca-F) [52,53,54]. This biological effect varies with species and fucoidan’s molecular weight [55]. Indeed, fucoidan of over 30 kDa or high concentration of LMWF may deplete the medium from growth factors and thus interfere with their activities [56,57]. This sequestration by fucoidan on growth factor could explain the inhibition of MMP-2 and -9 secretions.

Interestingly, in the absence of growth factors, cytokines or serum in the culture medium, fucoidan had no effect on the activation of the ERK 1/2 or p38 signaling pathways (data not shown). Being a glycosaminoglycan, fucoidan behaves as a heparin sulfate and binds to the cell surface. Fucoidan would appear to facilitate the interaction between MCP-1 and its receptor CCR2. We have previously demonstrated that fucoidan potentiates the activity of specific factors like FGF-2 on blood vessel formation *in vitro* [58] and *in vivo* [30]. Overall, our results help to explain the effects of fucoidan on monocyte adhesion and migration and support the therapeutic potential of fucoidan in chronic limb ischemia.

## 4. Experimental

### 4.1. Reagents

Fetal bovine serum, PBS −/−, HBSS +/+ and RPMI 1640 culture medium were from Gibco (Life Technologies, Saint-Aubin, France). Calcein-AM and PD98059 were from Calbiochem (Merck KGaA, Darmstadt, Germany). Giemsa, bovine serum albumin, gelatin and saponin were from Sigma Aldrich (Saint-Quentin-en-Yvelines, France). SB203580 was a kind gift from Bachelot-Loza (Inserm UMR_S 1140, Faculty of Pharmacy, Paris Descartes University, France). pERK was from Cell Signaling (Ozyme, Saint-Quentin-en-Yvelines, France), pP38 was from Promega (Lyon, France), αM-PE, α4-PE and CCR2-APC were from BD Biosciences (Le Pont de Claix, France), β1-FITC, ERK and GAPDH were from Santa Cruz (Heidelberg, Germany), β2 was from Chemicon-Europe (Merck KGaA, Darmstadt, Germany), and MCP-1 was from R&D systems (Bio Techn Lille, Lilie, France). LMW fucoidan was obtained by radical depolymerization of high-molecular-weight fucoidan extracted from *Ascophylum nodosum*, using procedures adapted from Nardella *et al*. [59]. The molecular weight average mass was 4 ± 1 kDa and characterized by high-performance steric chromatography (HPSEC) in 0.15 M NaCl, 0.005 M NaH2PO4 at pH 7.0, using two columns connected in series (Licrospher Si300 diol and Hema Sec Bio 40 columns) (Merck S.A., Molsheim, France) calibrated using narrow cut heparin fractions as described in Mulloy *et al*. [60]. The chemical composition was as follows: 34% fucose, 4% galactose, 3% xylose, 3% uronic acid and 32.2% sulfate. The human monocyte isolation kit II was from Miltenyi Biotec (Paris, France) and Histopaque solution from Sigma (Saint Quentin Fallavier, France).

### 4.2. Monocyte and HUVEC Isolation

Monocytes were isolated from healthy donor blood purchased from Etablissement Français du Sang (EFS, convention number: 13/EFS/064). Mononuclear cells were isolated by density-gradient centrifugation using 1.077 g/mL Histopaque solution and then negatively purified following the manufacturer’s procedure. Human umbilical vein endothelial cells (HUVEC) were isolated from cord blood with the mothers’ consent, as described by Zemani *et al*. [31].

### 4.3. Cell Adhesion Assay

Ten thousand monocytes were treated with fucoidan 10 μg/mL for 24 h and seeded on Millicell EZ slides (from Millipore, Merck KGaA, Darmstadt, Germany) coated with 0.2% gelatin. They were allowed to adhere for 30 min and then washed with PBS to detach non-adherent cells. Adherent cells were fixed with paraformaldehyde for 10 min at room temperature, then washed with PBS and permeabilized with 0.5% saponin. Cell nuclei were stained with TOPRO for 10 min. The slides were then coverslipped with Ibidi mounting medium and examined with a confocal fluorescence microscope.

### 4.4. Cell Migration Assay

Boyden chambers were used for migration assays with 8-μm pore-size inserts (BD Biosciences, Le Pont de Claix, France) in 24-well plates. Six hundred microliters of RPMI 1640 medium-1% FBS with 100 ng/mL MCP-1 was placed in the lower chamber. Seventy-five thousand monocytes treated with 10 μg/mL fucoidan were placed in the upper chamber in RPMI 1640 medium-0.1% BSA. After 4 h of migration, the inserts were fixed and stained with Giemsa (Sigma-Aldrich, Saint-Quentin-en-Yvelines, France). Migratory cells were counted in 10 randomly selected fields (200× magnification).

### 4.5. Transmigration Assay

HUVEC were seeded at 60,000 cells per Transwell chamber coated with 0.5% gelatin for 2 days. They were then activated for 4 h with 10 ng/mL TNFα, and 75,000 monocytes treated with 10 μg/mL of fucoidan were stained with 5 M calcein-AM at 37 °C for 20 min before being added to HUVEC. After 18 h of transmigration, the upper part of the insert was cotton-swabbed to remove non-migrated cells. The remaining cells were fixed, then the Transwell inserts were cut out, placed on slides and coverslipped with Ibidi mounting medium. The lower side of the insert was examined with a confocal fluorescence microscope. Labeled monocytes were counted in 10 randomly selected fields (200× magnification).

### 4.6. Western Blot

Total protein was prepared from monocytes treated with lysis buffer (Tris 50 mM, NaCl 150 mM, 1% Triton X100, PMSF 1 mM, Na3VO4 1 mM) supplemented with a protease and phosphatase inhibitor cocktail (Sigma Aldrich, Saint-Quentin-en-Yvelines, France) for 20 min on ice, then centrifuged for 10 min at 14,000× *g*. Supernatants were fractionated by SDS-PAGE 4%–12% (NuPAGE^®^ Bis-Tris Pre-Cast gels, Life Technologies, Saint-Aubin, France), transferred to nitrocellulose membranes, and incubated with the following primary antibodies: phosphor ERK, phosphor p38 and GAPDH (all at 1/300 in 0.1% milk/TTBS 1×) and then incubated for 10 min with SNAP i.d.^®^ (Millipore, Merck KGaA, Darmstadt, Germany). Secondary antibodies were either anti-mouse or anti-rabbit Dylight fluor 680 or 800 conjugated antibodies (Thermo Fisher Scientific, Villebon-sur-Yvette, France) (1/3000). Images of the blots were scanned with the Odyssey Infra-Red Imaging System (Li-Cor Biotechnology Eurobio, Courtaboeuf, France). Phosporylation was quantified with ImageJ software (National Institutes of Health, Bethesda, MD, USA).

### 4.7. Flow Cytometry

Monocytes treated with fucoidan for 30 min or 24 h were collected in HBSS containing 10% FBS. Cells were then labeled for 30 min at 4 °C with the following antibodies: αM-PE, α4-PE, CCR2-APC or β1-FITC. For β2 staining, cells were incubated for 30 min with anti-β2 then washed with HBSS–10% FBS and incubated with FITC-conjugated secondary antibodies for 30 min. Fluorescence was quantified in a BD Accuri C6 flow cytometer (BD Biosciences, Le Pont de Claix, France).

### 4.8. Zymography

One hundred five monocytes were seeded in 22.6-mm-diameter culture dishes starved overnight before being treated with fucoidan for 30 min. The culture supernatant was collected and 20 μL was analyzed as described by Sarlon *et al*. [30].

### 4.9. Statistical Analysis

Data are expressed as mean and S.E.M. Data were analyzed by one-way ANOVA followed by Turkey’s multiple comparisons test or Student’s *t* test. A *p* value < 0.05 was considered to denote statistical significance. GraphPad Prism software version Prism 5 (GraphPad, Sandiego, CA, USA) was used for all analyses.
